# In-Situ Monitoring of Reciprocal Charge Transfer and Losses in Graphene-Silicon CCD Pixels

**DOI:** 10.3390/s22239341

**Published:** 2022-11-30

**Authors:** Munir Ali, Yunfan Dong, Jianhang Lv, Hongwei Guo, Muhammad Abid Anwar, Feng Tian, Khurram Shahzad, Wei Liu, Bin Yu, Srikrishna Chanakya Bodepudi, Yang Xu

**Affiliations:** School of Micro-Nano Electronics, ZJU-Hangzhou Global Scientific and Technological Innovation Center, ZJU-UIUC Joint Institute, State Key Laboratory of Silicon Materials, Zhejiang University, Hangzhou 310027, China

**Keywords:** charge-coupled devices, charge transfer, multiplication, graphene

## Abstract

Charge-coupled devices (CCD) allow imaging by photodetection, charge integration, and serial transfer of the stored charge packets from multiple pixels to the readout node. The functionality of CCD can be extended to the non-destructive and in-situ readout of the integrated charges by replacing metallic electrodes with graphene in the metal-oxide-semiconductors (MOS) structure of a CCD pixel. The electrostatic capacitive coupling of graphene with the substrate allows the Fermi level tuning that reflects the integrated charge density in the depletion well. This work demonstrates the in-situ monitoring of the serial charge transfer and interpixel transfer losses in a reciprocating manner between two adjacent Gr-Si CCD pixels by benefitting the electrostatic and gate-to-gate couplings. We achieved the maximum charge transfer efficiency (*CTE*) of 92.4%, which is mainly decided by the inter-pixel distance, phase clock amplitudes, switching slopes, and density of surface defects. The discussion on overcoming transfer losses and improving *CTE* by realizing a graphene-electron multiplication CCD is also presented. The proof of the concept of the in-situ readout of the out-of-plane avalanche in a single Gr-Si CCD pixel is also demonstrated, which can amplify the photo packet in a pre-transfer manner.

## 1. Introduction

Photoexcited carrier integration and multiplication play a pivotal role in developing efficient photodetection, especially for weak incident light. Charge-Coupled Devices (CCD) and Complementary Metal-Oxide-Semiconductor (CMOS) are two leading imaging technologies with specific advantages and disadvantages. The attractive factors for using the CCD are its simple metal-oxide-silicon (MOS) photogate detector, which offers high sensitivity, high fill factor, and low noise. For CMOS imagers, the independent pixel structure provides random access, simple clocking, fast parallel readout, natural anti-blooming, and low power consumption [1,2,3,4]. Emerging imaging technologies require benefits from both CCD and CMOS architectures. Whereas all major imaging technologies based on CCD and CMOS often suffer from loss of image quality and contrast due to extensive transport and weak charge-to-voltage conversion efficiency.

Graphene can provide absorption tunability that can extend to UV to terahertz band by electrically adjusting the Fermi level without any additional manufacturing cost [5]. Unique properties of graphene open opportunities in various application areas, such as photodetectors, chemical sensors, and metamaterial absorbers that even extend to environmental cleanup functionalities [6,7,8] and protection units [9]. Graphene-based photodetectors often exhibit responsivity within 100 mA/W [10], resulting from short carrier recombination time (~2 ps) and recombination length (~1 μm), while the charge transport lengths in photodetectors are often extending to hundreds of microns. Thus, collecting photo-induced carriers before recombination within the graphene channel is still a challenging task [11,12,13]. One of the approaches to overcome this issue is integrating graphene with conventional device architectures like CCD and CMOS.

Graphene can work as an atomically-thin, transparent, charge-sensing layer that, by leveraging upon the photogating phenomenon through electrostatic capacitive coupling, detects absorption within a thicker adjacent substrate rather than serving as the light-absorbing medium. The changes in the graphene channel conductance can be sensed by the application of a constant bias voltage causing channel current $\left(I_{ds}\right)$. This current is proportional to the product of carrier density $\left(n\right)$ and mobility (*µ*) of graphene ($I_{ds}$ *∝* *µnE*). Moreover, graphene’s significant ambipolar mobility ($\sim 10^{3}-10^{5}\;{\mathrm{cm}}^{2}/V.s$) [14] provides a built-in photo gain mechanism that can enhance the detector response. For photo-gated graphene devices, responsivity higher than 1000 A/W has been reported [15,16,17,18]. This photo gating of graphene offers a solution to the previously mentioned issue by allowing a hybrid imaging scheme that adopts the benefits of both CCD and CMOS architectures while providing a direct readout of the integrated photoexcited charge and allowing flexible manipulation of the information signal (charge packet).

Previous works have demonstrated the potential of graphene-based CCD schemes for direct readout in a broad wavelength range from X-ray to mid-infrared (MIR) [19,20,21]. The next crucial step towards large-scale Gr-Si CCD imagers is to realize direct readout of charge multiplication and integration within the charge domain via graphene. A large number of serial transfers in CCD structure at high electric fields sufficient to trigger impact ionization leads to charge amplification within the charge domain before the charge-to-voltage conversion. In this work, we present the proof of concept for in-situ monitoring of the serial charge transfer between adjacent Gr-Si CCD pixels and investigate the potential of this platform to achieve charge multiplication in reciprocating neighboring pixels.

Moreover, the proof of the concept of the in-situ readout of an out-of-the-plane avalanche in silicon absorber through displacement ($I_{g}$) and graphene channel ($I_{ds}$) currents are explored as well in this work. The Gr-Si CCD pixel is initially dynamically ramped into deep depletion. At this stage, pulsed laser excitation triggers an avalanche effect via photoionization in the device that can be sensed by the two probe techniques discussed above.

## 2. Device Fabrication and Measurements

An n-doped $\mathrm{Si}/{\mathrm{SiO}}_{2}$ (500 µm/100 nm) substrate with a resistivity of $\left(1-10\right)\;Ω\cdot \mathrm{cm}$ corresponding to a doping concentration of $4.5\times 10^{14}{\;\mathrm{cm}}^{-3}\;\mathrm{to}\;4.94\times 10^{15}{\;\mathrm{cm}}^{-3}$ is used in this work. The ${\mathrm{SiO}}_{2}$ layer was patterned using photolithography with successive thermal evaporation (Angstrom Engineering) process to deposit Ti/Au film as the contact pads onto ${\mathrm{SiO}}_{2}$. The respective thicknesses of the Ti and Au metals are 5 and 70 nm. The chemical vapor deposition (CVD) graphene on a copper foil (ACS Materials) is first spin-coated with polymethylmethacrylate (PMMA) (ALLRESIST AR-26, speed =4000 rpm, time =60 s) [22]. The copper foil is then dissolved in a ${\mathrm{CuSO}}_{4}+\;\mathrm{HCl}\;+H_{2}O$ solution (${\mathrm{CuSO}}_{4}+\;\mathrm{HCl}\;+H_{2}O=10\;g:50\;\mathrm{mL}:50\;\mathrm{mL})$ for 5 h. Then the PMMA-coated graphene films are fished into the containers of deionized water and placed there for 2 h [23]. Then these films are transferred onto the top of the silicon wafer covering the pixel area and the metal electrodes.

Furthermore, PMMA was removed with acetone and cleaned with isopropyl alcohol (IPA). It was followed by the graphene patterning into square-shaped pixels, each having an area of $0.25{\;\mathrm{mm}}^{2}$, using photolithography and oxygen plasma. The photoresist was then removed by acetone and successive cleaning in IPA. Finally, wire bonding connects the top electrode with Au wires. Moreover, by using a Hitachi (Tokyo, Japan) S4800 field-emission microscope at an acceleration voltage of 5 kV, scanning electron microscopy (SEM) images are produced.

A dual-port signal generator provides gate voltage ($V_{g1}$ and $V_{g2}$) clocks. A power amplifier is used to generate large bias signals. The trans-impedance amplifier is implemented to measure the channel current ($I_{ds}$) of Pixel-1. The 532 nm wavelength laser is used for photo generation in the silicon absorber of the Gr-Si CCD pixel. The laser light is not always ON; instead, it is intermittent, with a frequency of 0.5 Hz having an ON duty of 2.5% for the charge transfer experiment.

## 3. Results and Discussion

### 3.1. Characterization of the Single Gr-Si CCD Pixel

**Figure 1:**
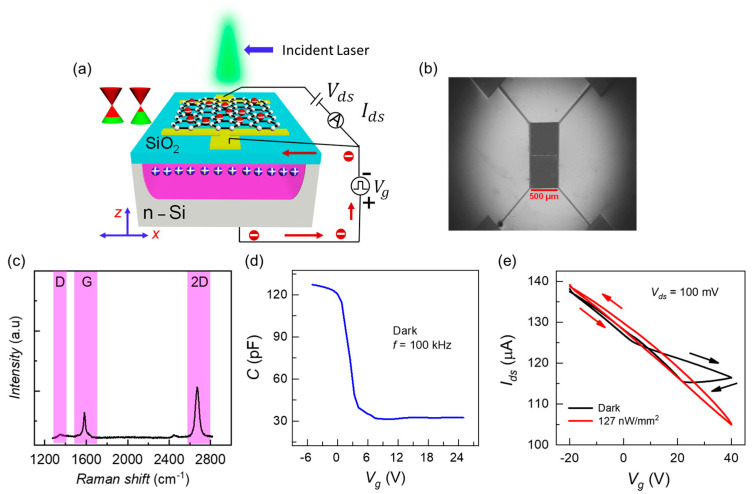
(**a**) A 3D schematic of Gr-Si CCD pixel, electrically connected to measure $I_{ds}$ and $I_{g}$. The biasing voltage $V_{g}$ and constant voltage source $V_{ds}$ are also included. (**b**) The SEM image of the two $500\times 500\;µm^{2}$ area Gr-Si CCD pixels platform. (**c**) The RAMAN spectra characterization shows the implementation of monolayer graphene as $I_{2D}/I_{G}>2$. (**d**) The quasi-static C-V measurement at 100 kHz modulation frequency in the dark scenario. (**e**) By quasi-statically sweeping the voltage $V_{g}$, the contribution of $\mathrm{Si}/{\mathrm{SiO}}_{2}$ and $\mathrm{graphene}/{\mathrm{SiO}}_{2}$ trapping states on hysteresis through $\left(I_{ds}-V_{g}\right)$ are observed within the operating voltages of the work. Both the forward and backward scans in the dark (black arrows) and under illumination (red arrows) are plotted.

The measurement scheme and essential device characterization of a single Gr-Si CCD pixel are presented in Figure 1. The 3D device schematic and the related electrical circuitry for measurements of $I_{g}$ and $I_{ds}$ are shown in Figure 1a. The scanning electron microscope (SEM) image of the dual Gr-Si CCD pixels platform with each graphene channel having an area of $500\times 500\;µm^{2}$ is shown in Figure 1b. Then, the Raman spectra of the graphene layer are shown in Figure 1c, the ratio of $I_{2D}/I_{G}$ is larger than two, emphasizing that the employed graphene is a monolayer. The monolayer graphene being highly tunable is a critical ingredient of our study.

The high-frequency capacitance-voltage (HF-CV) characteristics curve in the dark situation for gate voltage varying from −5 V to 25 V is shown in Figure 1d. The oxide capacitance ($C_{ox}$) of 126 pF in the accumulation state indicates the strong silicon dioxide layer required for the charge integration process. Before proceeding with the back-and-forth charge transport study, this characterization is essential for each of the Gr-Si CCD pixels.

Several device instabilities have been reported in graphene-based field effect transistors due to differences in graphene Dirac points, and the charge trapping effects in the substrate [24,25,26]. One such anomaly is the hysteresis in I–V characteristics [27,28] which originates from the materials involved and the operational environment [29,30]. The charge trapping in the underlying silicon substrate and ${\mathrm{SiO}}_{2}$ can contribute to this unwanted phenomenon. In addition, slow charge trapping at the $\mathrm{graphene}/{\mathrm{SiO}}_{2}$ interface produced by the contaminants from the wet transfer of graphene can also contribute to the hysteresis [31,32,33].

The $I_{ds}$ vs. $V_{g}$ curves during the forward and backward scans to probe the hysteresis effect in a single Gr-Si CCD pixel under dark and light conditions are shown in Figure 1e. A potential well in the silicon is compulsory to enable a Gr-Si CCD pixel for photodetection or to receive the laterally moving carriers. Hence, there are always some losses due to surface defects for the charge packet in the silicon well. Moreover, when surface defects are filled during light exposure, the vertical graphene-oxide-silicon structure behaves as a metal-insulator-metal capacitor. In this case, hysteresis will be mainly due to graphene, which is obvious from Figure 1e for laser ON condition. The constant optical illumination is intentionally kept large to cause inversion in the well so that the effect of $\mathrm{graphene}/{\mathrm{SiO}}_{2}$ traps on $I_{ds}$ can be subsided.

At 0 V gating bias, the Δ$I_{ds}$ due to hysteresis for the illumination condition is approximately 2 µA. Moreover, the nature of $\mathrm{Si}/{\mathrm{SiO}}_{2}$ interface trappings and $\mathrm{graphene}/{\mathrm{SiO}}_{2}$ interface defects is different as seen from the directions of arrows in the reverse bias scenario of silicon photogate for dark and optical illumination conditions. We used high-quality substrates from the UniversityWafer, Inc. (South Boston, MA, USA) in our experiments. The high-quality thermally grown ${\mathrm{SiO}}_{2}$ of 100 nm thickness does not contain excessive charge trapping.

These trappings are also not introduced due to excessive electric fields on ${\mathrm{SiO}}_{2}$ as our operating fields are within 4 MV/cm. Therefore, defects and trap states at the $\mathrm{graphene}/{\mathrm{SiO}}_{2}$ interface are the main contributing factors in the hysteresis of channel current rather than the underlying semiconductor substrate. Moreover, $\mathrm{graphene}/{\mathrm{SiO}}_{2}$ interface trapping states can be controlled by capping the graphene with an ${\mathrm{Al}}_{2}O_{3}$ dielectric layer [34].

**Figure 2:**
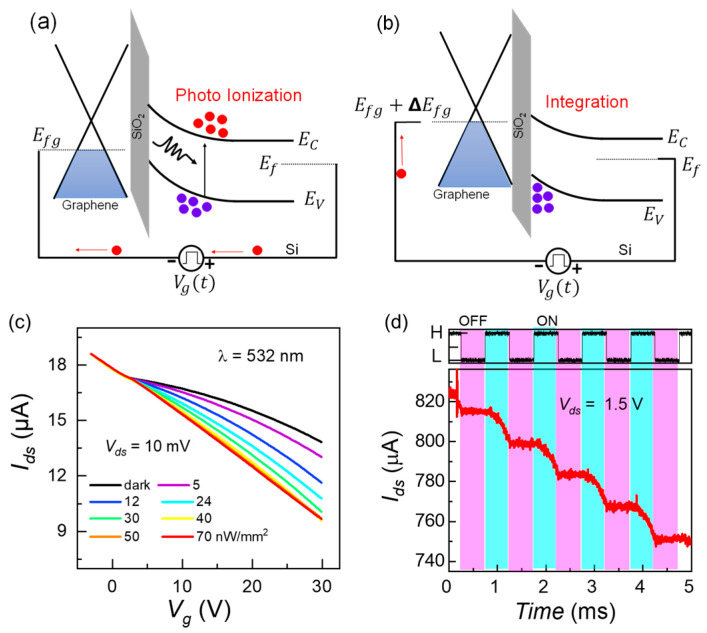
(**a**) Energy band diagram showing the photoionization process in the space charge region; it is equally valid for a constant or pulsed laser illumination. (**b**) This energy band diagram expresses that electrons drift into the bulk silicon and holes integrate at the $\mathrm{Si}/{\mathrm{SiO}}_{2}$ interface, causing a change in graphene’s Fermi level ($\Delta E_{fg}$). (**c**) The quasi-static $I_{ds}-V_{g}$ curves for multiple constant exposures of 532 nm wavelength laser. (**d**) The capability of the GCCD pixel for photo-sensing and multi-logic memory applications is illustrated using $I_{ds}$. For a square gate bias $V_{g}=33\;V$, $V_{ds}=1.5\;V$, and 532 nm laser pulses, charge integration can be monitored in real time by $I_{ds}-t$.

The band diagrams explaining the photoionization event and subsequent charge integration phenomena as the photo-ionized carriers transport to the $\mathrm{Si}/{\mathrm{SiO}}_{2}$ interface are shown in Figure 2a,b, respectively. The random-access mode, the unique feature enabled by graphene in the CCD structure, is observed by tuning the graphene Fermi level ($E_{fg}$) by the minority carriers accumulated at the underlying $\mathrm{Si}/{\mathrm{SiO}}_{2}$ interface of a Gr-Si CCD pixel. The integrated holes reflect graphene’s conductance variations while the electrons sink into bulk silicon.

Then the plausibility of the charge integration and readout within the single Gr-Si CCD pixel is confirmed through the creation of a potential well by quasi-statically reverse biasing the silicon substrate, followed by a 532 nm constant laser illumination and subsequent real-time sensing of integrated charges through $I_{ds}$ as shown in the p-type branch of the “V” shaped transfer curve in Figure 2c. For multiple laser intensities, we observed the changes in the Fermi level of graphene indicated by the increase in the slopes of the transfer curves. Here, the $I_{ds}$ decreased with increased laser power due to the rise in overall photoelectrons transferred to graphene to balance the photo holes in the deep depletion well. The observed photogating effect in the device should mainly come from the photo hole integration in the deep depletion well in silicon rather than the charge trapping in the oxide or the interface [28].

Moreover, due to several output current levels, the Gr-Si CCD pixel can be used as multilevel logic technology, as shown in Figure 2d. A square kind of $V_{g}$ signal of 1 kHz frequency and a high voltage level of 33 V is applied to the silicon semiconductor to construct a deep depletion state. Then, a 532 nm pulsed laser (50% duty) is multiply shined as shown in the top panel by black waveform. Charge integration (the stair-like waveform) is monitored in real-time through $I_{ds}$.

### 3.2. Back-and-Forth Charge Transfer for Multiplication

Charge multiplication occurs in CCD arrays when the applied gate electric field is large enough for impact ionization. Usually, a single impact ionization event is not strong enough to generate charge carriers to provide a high-quality image. Therefore, multiple impact ionization events are required to create a charge density sufficient to achieve significant image contrast. Back-and-forth serial charge transfer into a potential well is one such strategy for carrier multiplication.

**Figure 3:**
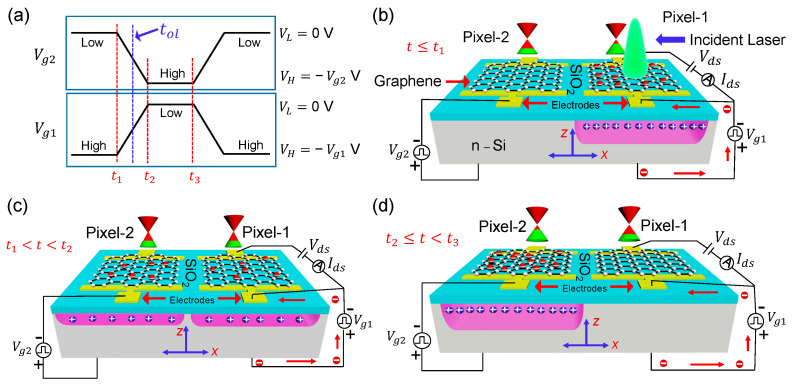
(**a**) The temporal dynamics of $V_{g1}$ and $V_{g2}$ phasing gate voltages are shown, indicating the crucial moments in the back-and-forth charge transfer experiment. (**b**–**d**) The schematics and related electrical circuitry required for the two adjacent Gr-Si CCD pixels for three conditions $t\leq t_{1}$ ($V_{g1}$ is high), $t_{1}<t<t_{2}$ (switching of $V_{g1}$ and $V_{g2}$), and $t_{2}<t<t_{3}$ ($V_{g2}$ is high) are shown. In-situ sensing of the back-and-forth charge transfer between neighboring pixels can be traced by the channel current $I_{ds}$. The charge density in the potential well affects the Femi level position in graphene. $V_{g1}$ and $V_{g2}$ induce potential wells in the adjacent pixels. The fringing electric field produced at the $\mathrm{Si}/{\mathrm{SiO}}_{2}$ interface in the inter-pixel region drives the charge transfer.

The silicon absorber is the common ground during the charge transfer experiment. At the same time, large negative potentials are applied to the two top graphene gates complementarily to create quantum wells inside the n-type silicon substrate. Crucial temporal points for the back-and-forth charge transfer by the square gating voltages of different switching durations for two consecutive pixels in a single clock cycle are displayed in Figure 3a. The schematics of the dual pixel platform and electrical circuitry to perform serial charge transfer using gate-to-gate-coupling and direct-readout by random-access mode in pixel-1 for three conditions $t\leq t_{1}$ ($V_{g1}$ is high), $t_{1}<t<t_{2}$ (switching of $V_{g1}$ and $V_{g2}$), and $t_{2}<t<t_{3}$ ($V_{g2}$ is high) are shown in Figure 3b–d, respectively.

The laser is exposed vertically to the Gr-Si CCD heterostructure from the top for all the optical measurements involved in this work. For the charge transfer experiment, laser light is not always ON; it is intermittent with a frequency of 0.5 Hz having an ON duty of 2.5%. Photoionization and successive charge integration in pixel-1 tunes $E_{fg}$, as shown in Figure 3b. The lateral fringing field at the $\mathrm{Si}/{\mathrm{SiO}}_{2}$ interface in the inter-pixel region is generated during the switching of $V_{g1}$, and $V_{g2}$. This fringing field drives the carriers in the two-dimensional hole gas (2DHG) of pixel-1 in the x-direction towards the depletion well of Pixel-2.

The scheme in Figure 3c shows an intermediate stage (of integrated photo holes for $t_{1}<t<t_{2}$) corresponding to the switching overlap moment ($t_{ol}=(t_{1}+t_{2})/2$), where the gates become equipotential. Figure 3d depicts that the entire hole population has been transferred into the quantum well of Pixel-2. Then, Pixel-1 retains its initial dark current state as the electrostatic doping of graphene vanishes. The first largest change of $I_{ds}$ belongs to holes-integration due to laser illumination. Then for all the charge-receiving events (backward charge transport from pixel-2 to pixel-1), $\Delta E_{fg}$ will be induced by laterally received carriers instead of photo-ionized carriers. Due to the losses during back-and-forth movement and interface state trapping losses, the overall $\Delta E_{fg}$ keeps decreasing.

During the photoresponse measurements, the laser is illuminated on a pixel enabled for the readout of the initial photoresponse. In addition, the in-situ readout is limited by the saturation of $I_{ds}$, as the maximum number of available states that can be electrostatically doped are limited in graphene. Shining the laser at large intensities generate higher number of photocarriers, leading to the saturation of $I_{ds}$ in pixel-1 and thus deviates from the linearity of the readout.

### 3.3. Carrier Losses during Back-and-Forth Transfer

Back-and-forth charge transfer between potential wells of adjacent pixels requires suppression of transfer losses such as interface state trapping loss, recombination loss, and losses due to backscattered carriers from the substrate. The interface trap losses can be removed by circulating a background charge [35], and introducing an offset in the driving clocks can reduce recombination losses [36]. As we experimented with a slow clock frequency (10 Hz), carrier backscattering at $t_{ol}$ does not affect measurements [37].

In our adjacent Gr-Si CCD pixels, pixel-1 functions as the photodetector and then, relying upon the random access mode, acts as the sense node (in standard CCDs, the sense node is a complete readout module consisting of many different elements). When the charge has been transferred from Pixel-1 to Pixel-2, $I_{ds}$ current retains its un-gated level (as no well exists beneath pixel-1, thermal or photo gating of top graphene is no more possible). The pixel-1, while working as the readout element, can sense every second transfer event, i.e., once photoionization happens in the pixel-1 corresponding to the maximum decrease of $I_{ds}$, its potential well demolishes for the transfer event from pixel-1 to pixel-2 and then enables again for the backward movement of the charge packet. The initial charge packet was transported twice before the subsequent readout. Moreover, $I_{ds}$ current for every receiving step (from Pixel-2 to Pixel-1) increases successively, indicating the presence of in-pixel interface state trappings and interpixel transfer losses from the substrate; these losses act twice on the charge packet before every readout.

The fraction of electrons that are successfully moved from one pixel to another is described by the charge transfer efficiency (CTE), while the loss fraction is calculated as 1-CTE. If $Q_{o}$ is the initial integrated charge and $Q_{n}$ is the charge that reaches the sense node (pixel-1 in our case) after n transfers, then $CTE^{n}=\frac{Q_{n}}{Q_{o}}$. In our case, for every readout event n=2. We measured four $I_{ds}$ traces for different amplitudes of $V_{g1}$ and $I_{ds}$ clock phases, as shown in Figure 3a.

**Figure 4:**
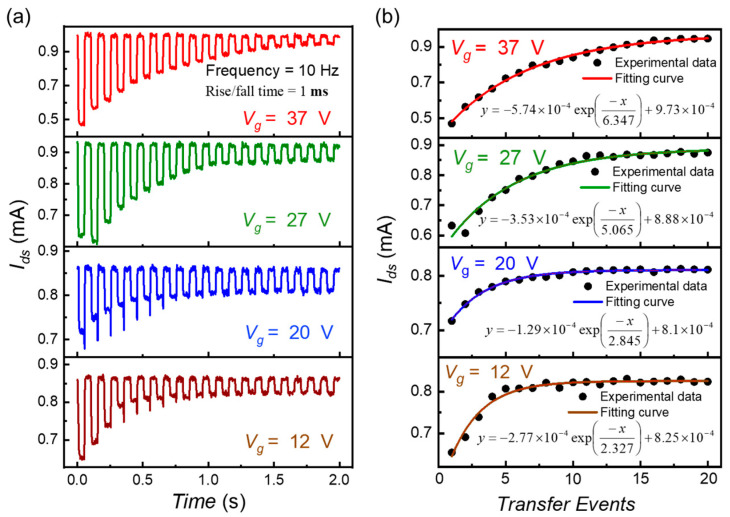
(**a**) For the back-and-forth charge transfer experiment for two adjacent Gr-Si CCD pixels platform, $I_{ds}$ is recorded when $V_{g1}$ and $V_{g2}$ phases are applied to the two graphene gates. The to and fro transfer of charges is shown for 20 cycles of two input phases. For almost all the amplitudes and switching slopes of two phases, a decaying trend in the initial $E_{fg}$ of the readout graphene channel is observed. This decay collectively corresponds to the in-pixel defect trappings and recombination losses in the inter-pixel region (**b**) The $I_{ds}$ increase with each readout, reducing the net number of charges. The fitting of the $I_{ds}$ values traces the losses. For four $V_{g1}$ $\left(12,\;20,\;27,\;37\right)\;V\;\mathrm{and}\;1\;\mathrm{ms}$ switching slopes, exponential fittings successfully highlight the transfer losses. The decaying exponential equations for each measurement situation are also mentioned.

For all the $I_{ds}$ curves shown in Figure 4a, exponential fittings are plotted in Figure 4b. The decay rates for $V_{g1}$ and $V_{g2}$ (12, 20, 27, 37) V are 1/2.327, 1/2.845, 1/5.065, and 1/6.347, respectively. Then the corresponding $CTE^{2}$ for all four situations are $e^{-1/2.327}$, $e^{-1/1.845},\;e^{-1/5.065}$ and $e^{-1/6.347}$. The square root will result in CTE’s of 0.806,0.838,0.906, and 0.924, respectively.

Parasitic capacitance often causes unwanted oscillations at the output of the circuit during the extended frequency operation of electronic devices. Whereas the operational frequencies of $V_{g1}$ and $V_{g2}$ reported in this work (10 Hz) are too low to cause any feedback loop. Another possible contribution of parasitic capacitance might originate from the proximity effect based on interpixel distance. However, the dimensions of our individual Gr-Si CCD pixels and corresponding interpixel distance (5 μm) are long enough to supress any such proximity effects to cause parasitic capacitance. Therefore, parasitic capacitance is not a dominating factor in our reciprocating charge transfer process.

**Figure 5:**
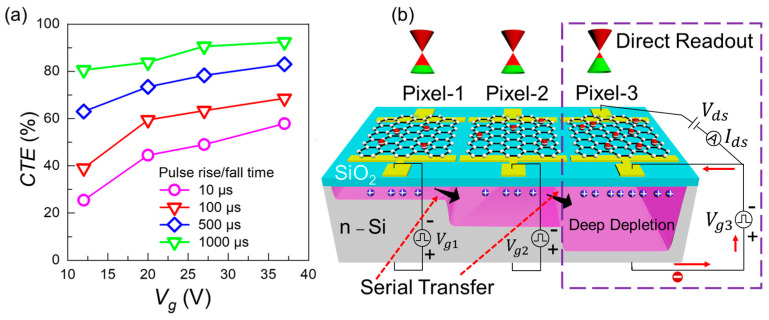
(**a**) CTE is plotted vs. phase amplitude at various switching slopes. (**b**) The 3D schematic for a three adjacent Gr-Si CCD pixels platform is shown with related circuitry to implement serial transfer and random-access modes due to $V_{g1}$, $V_{g2}$_,_ and $V_{g3}$ clock phases. Photo charges will be held in the left well; after strong fringing field development by ramping up, the charge will be released by removing the central barrier, and graphene will read the enhanced charge density by recording $I_{g}$ in the avalanche or multiplication unit.

In Figure 5a, CTE is plotted vs. phase amplitude ($V_{g}$) for switching durations of (10, 100, 500, and 1000) µs. The increasing fringing field and slow switching rates result in higher CTE’s. For almost all the gate bias values and switching slopes of two phases, a decaying trend in the first $\Delta E_{fg}$ of the readout graphene is observed. It shows the attenuation of the initial photo charge packet instead of any multiplication. We demonstrate that by exploiting random access and serial transfer modes together, the properties of CCD and CMOS technologies can be overlapped, allowing more flexible operation.

As transfer losses are unavoidable, controlled carrier multiplication can allow the lost carriers to boost CTE≥ 1. Reducing the inter-pixel distance will increase the fringing field. The minimum electrode separation attainable from photolithography is ~3–5 µm [1], while an undercut technique can reduce isolation up to ~0.1 µm [15], and electron beam lithography can further reduce the distance to 10 nm [38]. Moreover, factors like clock voltages integrated charges in the wells, and oxide thickness [17] also strongly influence the impact ionization probability.

To achieve carrier multiplication, electric fields larger than $\sim 10^{5}\;V/\mathrm{cm}$ are required [39,40,41]. In our dual-pixel Gr-Si CCD setup, the fringing field is enabled at $t_{1}$. The most probable moments for impact ionization are instantly after $t_{1}$ and before $t_{2}$, where fringing fields are the strongest. At the same time, a larger inter-pixel distance reduces the strength of the fringing field to reach the avalanche threshold. The large pixel sizes and higher density of 2DHG can presumably affect the multiplication in our device.

### 3.4. Device Scheme for Efficient Carrier Multiplication

We would like to propose a device scheme that can overcome the above-observed limitations and improve carrier multiplication in Gr-Si CCD pixels. The impact ionization becomes highly probable when minority carriers (holes) get a chance to move through strong enough fringing fields. In addition, carrier density should be low to avoid self-quenching, and multiplication can be significantly improved by providing sufficient acceleration to carriers before entering the fringing field.

The proposed device scheme uses three pixels as a single unit enabled by the serial charge transfer. The photo charges in these three adjacent MOS capacitors are temporarily held in the detector by a barrier until the avalanche unit is ramped to a strong fringing field. So, three clock phases are applied to three adjacent Gr-Si CCD pixels platforms (~1 µm distance). This platform can boost CTE and transfer speed benefitting from the random access and serial transfer modes. In Figure 5b, the 3D schematic and related circuitry are shown. The direct readout is implemented for the avalanche unit connected with $V_{g3}$, while $V_{g1}$ and $V_{g2}$ are associated with the detector and transfer units.

### 3.5. Out-of-Plane Avalanche Detection through Displacement and Channel Currents

**Figure 6:**
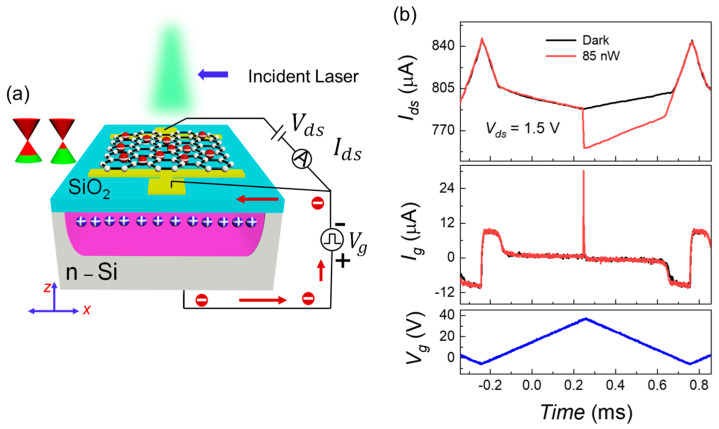
Displacement current ($I_{g}$) and channel current ($I_{ds}$) are two alternate readout probes for a single Gr-Si CCD pixel (**a**) The 3D schematic and related circuitry for measurement of out-of-plane avalanche (vertical to the $\mathrm{Si}/{\mathrm{SiO}}_{2}$ interface in the silicon potential well) through $I_{g}$ and $I_{ds}$ due to fast ramping $V_{g}$ is shown in Figure 5a. (**b**) Bottom panel: Fast ramping signal $V_{g}$ is demonstrated, and the laser pulse is shined just before the maximum ramp voltage to exploit the strongest surface potential ($\phi_{Si}$). Middle panel: The displacement current for light and dark conditions are shown. The rapid exponential increase corresponds to the photoionization event and subsequent avalanche. Top panel: The channel currents for light and dark conditions are shown. The quick reduction corresponds to the photoionization and following avalanche event.

The displacement current is not an appropriate readout probe for traditional CCDs having multiple rows and columns requiring multiple top-gating phases to transfer carriers from one pixel to the next until they reach the readout sense node. Contrary to that, the displacement current can be used for direct readout of an out-of-plane avalanche event (z-directed) from a single Gr-Si CCD pixel. Moreover, this out-of-plane carrier multiplication due to the electrostatic coupling of the $\mathrm{Si}/{\mathrm{SiO}}_{2}$ interface with the top tunable graphene channel can be in-situ readout through $I_{ds}$. The measurement scheme for recording the $I_{g}$ and $I_{ds}$ currents while silicon is dynamically biased is shown in Figure 6a. This measurement scheme is the same as the single-pixel characterization scheme shown in Figure 1a. The only difference is the application of a fast ramping biasing signal.

When a MOS capacitor is biased with a dynamic ramp $V_{g}$, the semiconductor experiences dynamically changing states, i.e., accumulation, depletion, and deep depletion [42,43]. The accumulation region through $I_{g}=C_{ox}\;dV_{g}/dt$ depicts the collection of majority carriers at the $\mathrm{Si}/{\mathrm{SiO}}_{2}$ interface, where $dV_{g}/dt$ is the voltage ramp rate. The formation of the potential well starts beyond flat band voltage; this marks the beginning of the depletion region. Then, $I_{g}=C\;dV_{g}/dt$ as $C_{ox}$ is replaced by the differential capacitance $\left(C\right),$ which is the series combination of $C_{ox}$ and the semiconductor capacitance $\left(C_{Si}\right)$.

The $I_{g}$ and $I_{ds}$ currents are related to each other through $\frac{\partial I_{ds}}{\partial t}=I_{g}\frac{\mu V_{ds}}{L^{2}}$, where L is the pixel length. The correlation between these two currents in the dark and pulsed laser illuminations when silicon is ramped at fast pace (88 kV/s) is shown in Figure 6b. When the silicon absorber substrate is biased into accumulation (0 V to −6 V and −6 V to 0 V), the positive charge induced in the initially p-doped graphene is calculated by modeling the Gr-Si CCD heterostructure as an ideal parallel-plate capacitor.

During ramp-up (ramp-down), the quadratic decrease (increase) of $I_{ds}$ is directly related to the linear decrease (increase) of majority carriers (electrons) concentration at the $\mathrm{Si}/{\mathrm{SiO}}_{2}$ interface where $I_{g}=C_{ox}\;dV_{g}/dt$. Whereas maximum $I_{ds}=847\;µA$ at $V_{g}=-6$ V corresponds to the sum of the graphene’s maximum electrostatically doped and chemically doped holes, as shown in the top panel of Figure 6b. Then, this trend transitions in the depletion region (0.5 V to 5 V). Finally, space charge region donors in the deeply depleted well are reflected on graphene through electrostatically induced electrons;

The slow decreasing trend corresponds to a quadratically induced well where minimum $I_{ds}=791\;µA$ at $V_{p}=38\;V$ corresponds to the maximum $\phi_{Si}$.

The pulsed laser (930 nm) is illuminated slightly before the ramp-up to ramp-down transition. Which benefits from the maximum $\phi_{Si}$ of the strongly depleted space charge region to study post-photoionization and pre-integration avalanche events. The quick increase in $I_{g}$ and decrease in $I_{ds}$ belong to photoionization and successive multiplication for the pulsed laser-ON event. The sharp decrease in $I_{ds}$ corresponds to a transient surge of hole integration at the interface. Then, by offering a negative feedback loop, the self-regulating phenomenon produces screening, forcing the change of $\phi_{Si}$ to add with the voltage drop across oxide to maintain current continuity [44,45,46,47].

In our future work, we aim to systematically study a pre-transfer, out-of-plane avalanche in one pixel, which can help mitigate the transfer and defect trapping losses in the dual adjacent pixel platform along with the 3-phase measurement scheme shown in Figure 5b.

This work demonstrates the serial charge transfer in Gr-Si CCD for the first time. There is no previous work that we can directly compare. Since Gr-CCD is still at the initial prototype stage, it would not be appropriate to directly compare it with the functional parameters of commercial CCD devices. However, we would like to highlight the structural, fabrication, and operational advantages and limitations of the proposed device scheme with the traditional CCD structure in the below Table 1.

The small thermal carrier generation rate ($G_{th}$) of $312\;e/{\mu m}^{2}.s$ in silicon at room temperature has been reported, resulting in the inversion of the interface in ~200 s [20]. Thus, the contribution of thermal charge for charging our Gr-Si CCD pixel is almost negligible compared to the levels of illuminating powers employed. The sophisticated and high-performance CCDs display a one-half decrease in the dark current for every 5 to 9 degrees Celsius when cooled below room temperature, called doubling temperature. The CCDs are only required to operate at very low temperatures when their core purpose is to sense the far infra-red (FIR) energy photons, i.e., the James Webb space telescope is designed to operate at the lowest temperature up to −266 degrees Celsius, which enables it to record the past of a galaxy living at the far edge of the known universe. In our Gr-Si CCD photodetector, lowering the temperature will not help detect FIR wavelengths due to silicon’s large intrinsic band gap (1.12 eV) absorption limit. Increasing the temperature will boost the dark current level, which is also an unwanted aspect.

The customized measurement setup for charge transport will likely induce frequent graphene burnouts, oxide leakage, and tunneling as the complementary gating phases $V_{g1}$ and $V_{g2}$ are directly connected to graphene channels, particularly when in-situ readout loops of both pixels are enabled. The existing measurement setup is not that sophisticated to measure the transferring of integrated holes through the $I_{ds}$ of both pixels. The specific measurement setup offering multiple pulse modes and providing a good common ground is required. Moreover, it should be able to keep the alternating variation of gating phases from penetrating the $I_{ds}$ loop, which is an intrinsically direct current (DC) and recorded by an oscilloscope that is electrically fed through an uninterruptible power source (UPS). The simultaneous measurement of the transferring events along the x-direction through both pixels is also enlisted in our future works.

## 4. Conclusions

The random access and serial transfer modes are implemented to demonstrate the in-situ monitoring of the serial charge transfer process in a reciprocation platform composed of two adjacent Gr-Si CCD pixels. Due to excessive losses during reciprocating transfers, the maximum CTE is limited to 92.4%. The charge transfer efficiency intrinsically depends on factors like inter-pixel distance, phase clock amplitudes, switching slopes, oxide thickness, semiconductor doping density, and density of surface defects.

We also propose a scheme of three consecutive pixels for charge multiplication, as the fringing field can be enhanced by ramping up the avalanche unit before releasing the held carriers. The simultaneous application of two modes enables us to benefit from CCD and CMOS technologies altogether in one situation allowing flexible manipulation of charge packet. The out-of-plane multiplication is also demonstrated for a single Gr-Si CCD pixel, which can help overcome post-integration and pre-transfer losses.

## Figures and Tables

**Table 1:** The comparison between conventional silicon-based CCDs with novel Gr-Si CCD pixels is presented for different parameters, i.e., readout techniques, response time, cost of fabrication, and charge transfer efficiency.

Key Parameters	Gr-Si CCD	Silicon-Based CCD
Direct Readout	Yes	No
Broadband Response	Yes	No
Room Temperature Sensitivity	Higher ($\sim 6\times 10^{4}\;A/W$)	Lower
Readout time	$\sim \left(0.5–10\right)\;\mathrm{ns}$	~100 μs to 10 s [48,49]
Cost of implementation	Low	High
Response time	$\sim \left(0.5–10\right)\;\mathrm{ns}$	$\sim \left(0.5–10\right)\;\mathrm{ns}$
*CTE*	92.4%	99.999% [50,51,52]
Readout & Integration	Independent & Non-destructive	Dependent

## Data Availability

The data related to this study is available from the corresponding authors upon reasonable request.
